# Disease burden of methylmercury in the German birth cohort 2014

**DOI:** 10.1371/journal.pone.0190409

**Published:** 2018-01-11

**Authors:** Julia Lackner, Michael Weiss, Christine Müller-Graf, Matthias Greiner

**Affiliations:** 1 Department of Exposure, Federal Institute for Risk Assessment, Berlin, Germany; 2 Institute for Food Quality and Safety, University of Veterinary Medicine, Foundation, Hannover, Germany; Chinese Academy of Sciences, CHINA

## Abstract

This study aimed to estimate the disease burden of methylmercury for children born in Germany in the year 2014. Humans are mainly exposed to methylmercury when they eat fish or seafood. Prenatal methylmercury exposure is associated with IQ loss. To quantify this disease burden, we used Monte Carlo simulation to estimate the incidence of mild and severe mental retardation in children born to mothers who consume fish based on empirical data. Subsequently, we calculated the disease burden with the disability-adjusted life years (DALY)-method. DALYs combine mortality and morbidity in one measure and quantify the gap between an ideal situation, where the entire population experiences the standard life expectancy without disease and disability, and the actual situation. Thus, one DALY corresponds to the loss of one year of life in good health. The methylmercury-induced burden of disease for the German birth cohort 2014 was an average of 14,186 DALY (95% CI 12,915–15,440 DALY). A large majority of the DALYs was attributed to morbidity as compared to mortality. Of the total disease burden, 98% were attributed to mild mental retardation, which only leads to morbidity. The remaining disease burden was a result of severe mental retardation with equal proportions of premature death and morbidity.

## Introduction

Methylmercury is regarded as one of the most toxic mercury compounds. It predominantly arises from the methylation of inorganic mercury by the action of microorganisms in aquatic systems. Fish and marine mammals ingest methylmercury with their food and fish additionally absorb it through the gills. Methylmercury accumulates in the marine food chain. Overall, the highest methylmercury levels are found in predatory fish and big, old marine mammals [1, 2].

For humans, fish and seafood consumption is the most important exposure pathway. After the consumption of fish or seafood, methylmercury is absorbed in the intestinal tract and can pass the blood-brain barrier and also enters the placenta. Depending on the consumed quantities, damage to brain or central nervous system may occur [3, 4]. Especially the brain of the unborn child is vulnerable, so that fetal brain lesions are possible without signs of maternal intoxication [5]. In contrast to the brain damage of exposed adults, the fetal brain lesions are not locally restricted but are diffuse and can lead to symptoms such as cerebral palsy, mental disabilities, disturbances of senses, movement, speech or coordination disorders [6]. Little is known about the quantifiable effects of methylmercury, because of the various forms of symptoms caused by prenatal methylmercury exposure. Therefore this study would like to address this question. Cognitive deficits are often approximated by IQ point deficits. The exposure of unborn children can be estimated via levels of methylmercury in cord-blood, blood and hair of pregnant women or indirectly via the fish and seafood consumption of pregnant women together with the expected contamination levels of this food source [7]. To link the maternal exposure and the IQ point deficits of children, a dose-response relationship was estimated by Axelrad et al. (2007). Based on three epidemiological studies, Axelrad et al. (2007) estimated, that each μg/g mercury increase in hair of mothers, leads to a 0.18 point decrease in the individual IQ levels of their infants [8].

A well-established summary measure to rank the health impact of foodborne contaminants is the disability-adjusted life years (DALY), which combine morbidity and mortality [9].

Until now, there is only one study from the Netherlands, which calculates the disease burden of methylmercury due to fish consumption using the DALY-method. This risk-benefit study considered the health outcome IQ reduction and focused on two consumption scenarios (weekly fish intake of 200g or 500g) [10].

The aim of our study is to quantify the overall disease burden of methylmercury, ingested with fish or seafood, for Germany using empirical data on the weight of pregnant women, the amount of fish consumption and the methylmercury concentration of the consumed fish species. By using these empirical data in combination with Monte Carlo simulations, we wanted to develop a way to calculate the disease burden for contaminants with a highly variable concentration like methylmercury in fish and seafood.

## Methods

To achieve the study aim, we assessed in a first step the incidence rate of health impairment attributed to prenatal methylmercury exposure by integrating the available information via Monte Carlo simulation. In a second step, we used these computed values to quantify the burden of disease in terms of DALYs for the health outcome mental retardation, which we considered to be the most reliable and quantifiable outcome. To account for variability and uncertainty of model input quantities, a probabilistic analysis and scenario analysis was considered.

The following factors were estimated:

1. Dietary exposure of pregnant women
2. Incidence of mild or severe mental retardation
3. Resulting DALYs for these health endpoints.

The disease burden of methylmercury was estimated based on the methodological suggestions of the World Health Organization (WHO). As a nonlethal health outcome the WHO established the mild mental retardation (MMR), which is defined as IQ points ranging between 50 and 69 to assess the burden [7]. The maternal exposure levels were examined in relation to the IQ values of the children. To link the exposure levels and the IQ values, we used the maternal hair levels, because they are the best marker for organic mercury. The WHO implemented an approach for examining the relation of maternal hair mercury levels with the IQ level decrease in mother’s subsequently born children [1, 7]. We followed the assumption, that there is no threshold for the dose–response relationship and assumed that every intake of fish or seafood leads to an IQ reduction, because all analyzed fish and seafood samples showed some methylmercury concentration [3, 8, 11].

The incidence of mental retardation was defined as the number of children in the birth cohort, in whom the IQ level was shifted from a normal value to an IQ value under 70 points [7]. Shifts, which result in a postnatal IQ above 70 IQ points, are not considered, because these individuals seldom require a special medical treatment or education program. Furthermore, 70 IQ points is a widely accepted cutoff-point for mild mental retardation [12]. We extended the WHO model by adding the severe mental retardation (SMR) as a second health outcome. SMR is defined as an IQ value less than 50 points. With an IQ less than 50 points the morbidity increases and SMR is associated with premature mortality [13].

**Fig 1:**
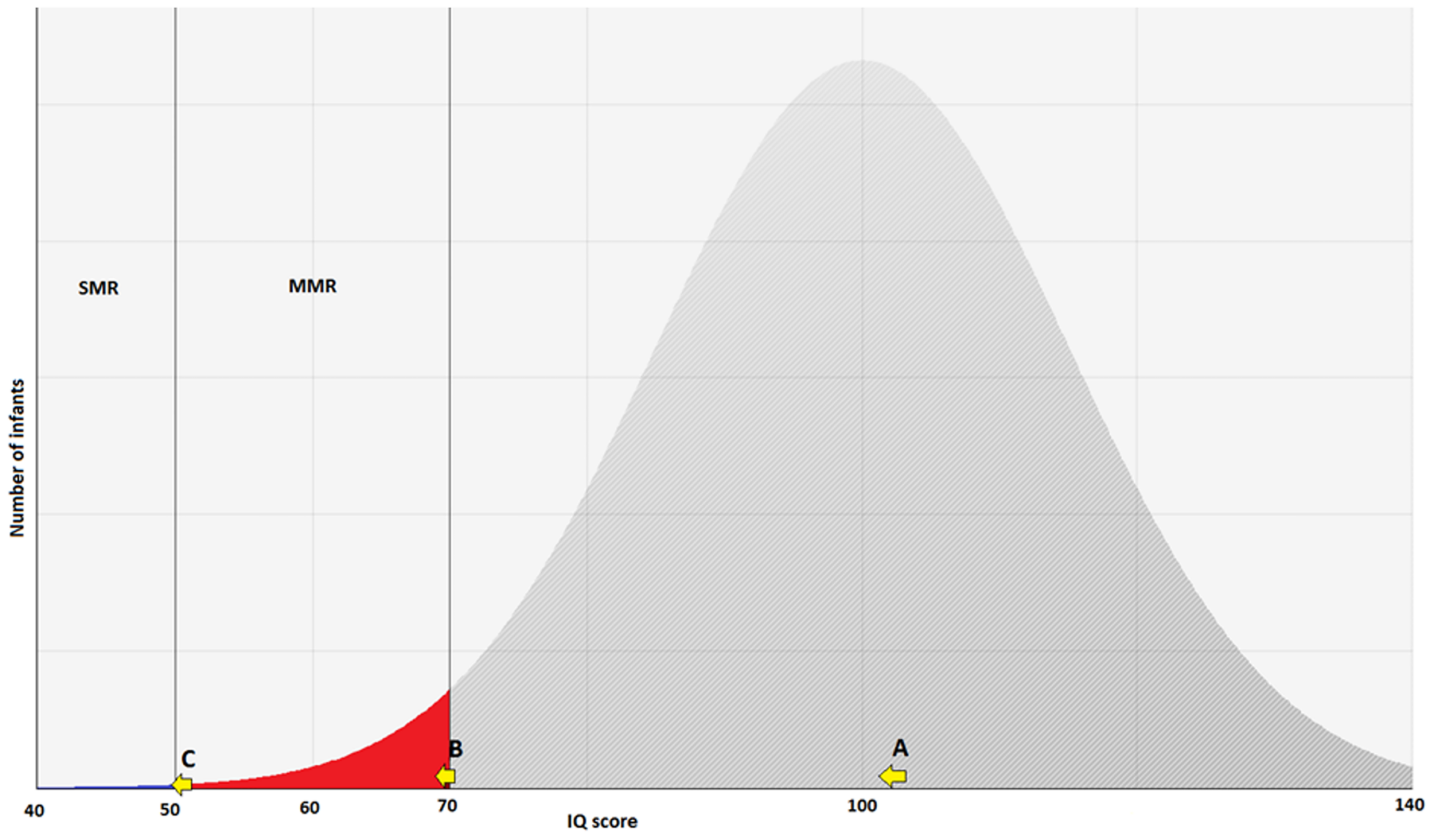
IQ-Shift of three methylmercury exposed children, whose mothers have eaten fish. The amount of absorbed methylmercury leads to a decrease in IQ points, which results in a disease burden for child B and C. Child A: no relevant retardation. Child B: mild mental retardation (MMR). Child C: incidence of severe mental retardation (SMR) modified after [7].

Fig 1 shows the effect for three children (A, B, C), whose mothers have eaten fish or seafood. The amount of absorbed methylmercury leads to a decrease in IQ points, which results in a disease burden for child B and C.

### Maternal exposure

To estimate the maternal exposure, we used the following intake model to convert the data of fish and seafood consumption to the level of methylmercury concentration in blood [14]:

$$C=\frac{d*A*f*bw}{b*v}$$

*C* = concentration in blood (μg/L)

*d* = exposure dose (μg/kg bw/day)

*A* = fraction of the dose absorbed (0.95)

*f* = absorbed fraction distributed to blood (0.05)

*bw* = body weight of pregnant women (kg)

*b* = elimination rate constant (0.014 per day^-1^)

*v* = blood volume (bw*0.065L/kg)

The exposure dose*d* was estimated by using the formula [15]:

$$d=\frac{fa*mc}{bw}$$

*fa* = amount of daily fish intake (g/day)

*mc* = methylmercury concentration in fish and seafood (μg/g)

For the fish and seafood consumption, we used the data from the German National Consumption Study (Nationale Verzehrsstudie (NVS) II), which includes 82 pregnant women [16–18]. This study contains the latest available consumption data for Germany, which were collected 2005–2006. Additionally, this study contains the body weights and the amounts of fish or seafood consumption [19]. Since the species of fish were not recorded in the consumption data, we applied the sale data for fish species in the Germany food market 2014 under the assumption that the relative consumption is equivalent in pregnant women [20]. Due to their specific foraging behavior and lifespan the different fish and seafood species show different levels of methylmercury [11]. To calculate the exposure, we used the data of two studies, which measured the methylmercury concentration in different fish species in the year 2008 [3, 11]. For each fish species, we built a beta-PERT distribution to describe the species specific methylmercury concentration[21]. For the minimum and maximum, we used the lowest and highest measured value reported respectively in either study.

The most likely value represents the median of the tested fish. Some fish species (e.g. herring) were examined in both studies. For the estimation of the most likely contamination level, we used the study with the highest number of tested fish [3, 11]. For fish species that are consumed in Germany, but for which data on methylmercury concentrations were not available, we used the mercury concentration from monitoring data (catfish) or used data from similar fish species [22]. Of the fish species, 7.4% were not classified. For this group “other species”, we applied the combined minimum, maximum and median (as most likely) value of all species of fish. The parameters for the beta-PERT distributions and the relative consumption of the different fish species are shown in S1 Table.

The fish concentration data were combined with the daily amount of fish intake. According to the NVS II, pregnant women (non-fish consumers excluded) eat on average 27.1g fish or seafood per day (standard deviation SD: 24.9g) and have a bodyweight of 70.3kg (SD: 13.1kg)[23]. We assumed that the consumption and the body weight are normally distributed. The consumption distribution should contain fish consumers exclusively and was therefore truncated, at a daily intake of 1g. Additionally, we set a maximum of 500g intake per capita and day. For the body weights of pregnant women, we truncated the normal distribution at 38.3kg (lowest body weight for women in the NVS II) and 200kg (highest body weight for women in the NVS II was 185kg plus normal weight increase in the pregnancy) [18, 23]. The incidence rate of MMR and SMR, was estimated by Monte Carlo simulation in R (Version 3.3.1, r-project) by using the mc2d package [24, 25]. We simulated a population of 643,435 persons. This number corresponds to the number of newborns in 2014, which survived birth (minus 10% for non-fish consumers under the pregnant women). This is the assumed absolute number of exposed children in the birth cohort 2014 in Germany [23, 26].

By using the information about maternal body weight*bw*, consumed fish amount *fa* and methylmercury concentration*mc*, we obtained distributions describing the methylmercury dose *do*, which allowed us to calculate the concentration in the maternal blood*C*.

For a further use of the WHO implemented dose-response model, it was necessary to convert the computed blood concentration *C* into a hair concentration. For this conversion we used the recommended factor of 1:250 from blood to hair [7]. In addition, we performed a Spearman′s rank correlation with R to show the influence of the various factors (amount of fish consumption, fish species and maternal body weight) on the hair values [25].

We followed the assumption that each μg/g mercury increase in hair of mothers, leads to a 0.18 point decrease in the individual IQ levels of their infants [8]. The incremental approach of the WHO, divides the maternal hair concentration into 2 μg/g intervals ranging from 0 to 100 μg/g. The IQ reduction uses the midpoint of each interval. The affected children were determined by simulation as those individuals in whom the methylmercury induced IQ point decrease resulted in a drop of the values below the thresholds of 50 or 70 IQ points. For example, in cases where the maternal hair concentration of methylmercury was 4 μg/g, the 2–4 μg/g interval was used, resulting in a 0.54 IQ point decrease (3 x 0.18 = 0.54) [7]. In combination with the IQ thresholds, this means that children with IQ points ranging from 70 to 70.54 points would shift from the ‘normal’ to the MMR area. Equally exposed children with IQ points ranging from 50 to 50.54 points would shift from the MMR to the SMR area.

As individual IQ base level, we used random numbers drawn from a normal distribution with a mean of 100 and a standard deviation of 15 [2, 7]. These IQ levels were linked to the simulated maternal hair values of 643,435 persons. Then, we counted the number of children whose IQ points shifted to SMR or MMR. Using these two thresholds, it was possible to determine one incidence estimate of SMR and one incidence estimate of MMR pertaining to the virtual birth cohort.

At this stage of the procedure, the inter-individual variability is reflected in the single-value incidence estimation. This procedure was repeated 1000 times to account for uncertainty in the maternal body weight, the amount of fish eaten and the consumed fish species with the corresponding methylmercury concentration to obtain the incidence rate of SMR and MMR in terms of a statistical distribution.

### DALY calculation

The burden of disease caused by SMR and MMR were summarized in DALYs, following the methodology proposed by Murray and co-workers [27, 28]:

DALY=YLD+YLL

YLL are defined as the number of Years of Life Lost due to mortality of a specific health outcome. The calculation is based on the number of all fatal cases (*d*) due to the health outcome(*l* = SMR or MMR) multiplied by the remaining life expectancy in years (*e*) [29]. The expected lifespan was derived from the life-table 2012/2014 reported by the German Federal Statistic Office. Female and male life expectancy was set to 83.05 and 78.13 years, respectively [30].

$$\text{YLL}=\sum_{l}d_{l}\times e_{l}$$

YLL were only calculated for the outcome SMR. To estimate the mortality and the average age at death, we conducted a literature review. In the last ten years the life expectancy of persons with SMR increased by 10 years [13]. We suspect that this positive trend will continue and assumed a uniform distribution of *d*with a minimum of 0 (no premature mortality) and the number of SMR incidence from our Monte Carlo simulation as maximum (all SMR affected children are dying earlier). We separately considered the average age at death for men and women in two beta-PERT distributions. The minimum value for both sexes was set at 58.6 years and the maximum value was taken from the life-table 2012/2014 for Germany [30, 31]. The most likely value was calculated based on Patja et al. (2000). Here, for IQ levels from 0–19 points a reduction of life expectancy over 20% for almost all ages was observed [32]. According to this study, we subtracted 20% of the life expectancy of the men and women for 2012/2014 resulting in most likely life expectation values of 62.5 years in men and 66.44 years in women (S2 Table).

YLD are defined as the number of Years Lived with Disability. The calculation is based on the incidence of a health outcome (*n*) multiplied by the duration (*t*) and the disability weight (*w*) of this illness [29]:

$$\text{YLD}=\sum_{l}n_{l}\times t_{l}\times w_{l}$$

Mental retardation is a congenital and lifelong disability. We used the recommended fixed value of 0.361 for the disability weight (*w*) of MMR [7]. For the *w* of SMR, we used the described span for mental retardation of the Global Burden of Disease (GBD) 2004 study and translated the parameters into a beta-PERT distribution with 0.402 for the minimum, 0.459 for the most likely and 0.484 for the maximum value [33]. To get exclusively the burden caused by methylmercury intake, we subtracted the MMR-disability weight from these three values. All of the children shifting in the SMR group would have been born with the burden of MMR, irrespective of the methylmercury consumption of their mothers, because the maximum loss of IQ points from the exposure model was 18 IQ points by a maternal hair concentration over 100 μg/g [7].

All input parameters are shown in S2 Table. The DALY calculation was performed with 100,000 iterations in R (Version 3.3.1, r-project) with the DALY Calculator [34]

### Sensitivity analyses

Several sources contributed to the variability and uncertainty of the estimation of disease burden. Due to limited information about the fish consumption in pregnant women, we investigated the uncertainty in three different scenarios. Here, we compared the above presented result (reference scenario) with three scenarios. In the first scenario pregnant high fish consumers were considered (85.4g per day with equal methylmercury concentration as in the reference scenario), the second and third scenario used the normal fish intake of pregnant women (27.1g per day) with the lowest (pollack: 0.001 μg/g) and highest (tuna 2.265 μg/g) measured methylmercury concentration. For the two last scenarios, we assumed constant intake at the indicated concentrations.

Additionally, we performed uncertainty analysis with the recommended values of the WHO [7]. We estimate the burden of methylmercury with a threshold of 0.94 or 2.15 μg/g mercury in maternal hair, with different dose-response relationships (0.012 or 0.387 loss of IQ points per μg/g mercury in hair) and with blood-hair conversion factor from 140 and 370.

The threshold values of tolerable methylmercury intake were obtained from the publications of the WHO and the United States Environmental Protection Agency (US EPA) [7]. We translated the published values in a first step into tolerable blood concentration values and finally into threshold values for methylmercury concentration in the hair following the method described above in the maternal exposure section. Methylmercury concentrations in the hair below these threshold values indicate that no disease burden should be expected. The values of the dose-response relationship correspond to the 95% confidence interval of the estimation from Axelrad et al. (2007) and blood-hair conversion values show the ranging in the literature [7, 8]. One study, which compared the results of the dietary model with measured data, received 14 times higher estimated values [35]. To show the uncertainty of the dietary intake model, we divided the output by 14 [7].

## Results

In a first step, we present the results of the incidence rates of MMR and SMR. In a second step, we show the calculated DALYs.

### Incidence mental retardation

Distribution parameters of simulated maternal hair and blood methylmercury concentrations are shown in Table 1.

**Table 1:** Calculated maternal hair and blood concentration of methylmercury.

	Mean	SD	Min	2.5%	25%	50%	75%	97.5%	Max
**Blood**	3.46μg/l	2.68μg/l	0.0249μg/l	0.269μg/l	1.53μg/l	2.83μg/l	4.68μg/l	10.3μg/l	32.1μg/l
**Hair**	0.865μg/g	0.67μg/g	0.00622 μg/g	0.0672 μg/g	0.383 μg/g	0.708 μg/g	1.17 μg/g	2.58 μg/g	8.03 μg/g

SD = Standard deviation; Min = Minimum; Max = Maximum; 2.5% -97.5% = Percentile

**Fig 2:**
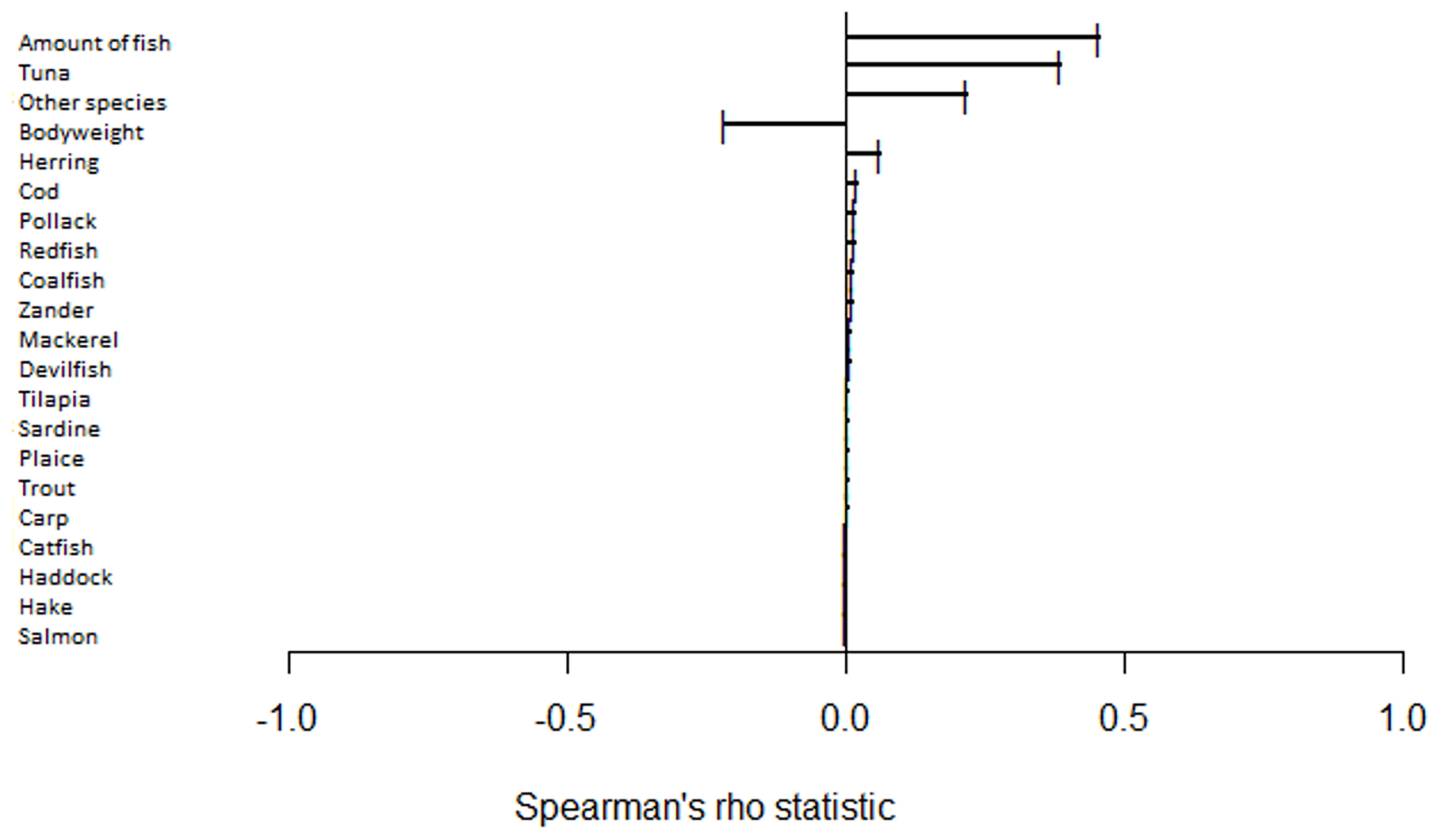
Tornado chart of the major influencing factors. Illustrating Spearman′s rank correlation coefficients of the major influencing factors and the maternal hair concentration of methylmercury.

Furthermore, we identified the major factors of influence of the maternal hair concentration with the Spearman′s rank correlation coefficient. We found a moderate positive correlation (0.45) for the amount of fish and a weak positive correlation for the consumption of tuna (0.38) and other fish species (0.22). Additionally, we found a weak negative correlation with the maternal bodyweight (0.22) (Fig 2).

Based on the hair concentration of methylmercury, we calculated an average incidence of 0.7529 with a standard deviation 0.035 for MMR per 1,000 exposed newborns. For SMR, we calculated an average incidence of 0.0218 with a standard deviation of 0.0058 per 1,000 exposed newborns.

### DALY calculation

The methylmercury-induced burden of disease for the German birth cohort 2014, ranged from 12,915 DALY (2.5th percentile) to 15,440 DALY (97.5th percentile) and had an average of 14,186 DALY. The distribution of the overall methylmercury-associated disease burden is shown in S1 Fig.

Our model suggest that 500 children of the 2014 birth cohort in Germany (714,927 children) shifted to the MMR or SMR area. 98% of the disease burden was caused by MMR. MMR only produce morbidity, which is described as YLD. In contrast, the disease burden of SMR was with 232 DALY substantially lower and corresponded to 2% of the overall DALYs. More than half of the SMR disease burden was associated with mortality (S2 Fig). 7 of the 14 SMR-cases lead to premature mortality (on average 18 years earlier than the not affected population).

Table 2 shows the total disease burden for the birth cohort 2014 and the burden for the same year split into the health outcomes SMR and MMR. For international comparison standardized data (DALY per 100,000 inhabitants and born children) are also reported.

**Table 2:** Disease burden of methylmercury for 2014 born children, per 100,000 born children and inhabitants.

	DALY for the birth cohort 2014	DALY per 100,000 born children	DALY per 100,000 inhabitants [36]
**Total DALY**			
**DALY**	14,186	1,984	18
**YLD**	14,057	1,966	17
**YLL**	129	18	0.16
**Cases**	494	69	0.62
**Deaths**	7	1	0.01
**MMR**			
**DALY**	13,954	1,952	17
**YLD**	13,954	1,952	17
**YLL**	0	0	0
**Cases**	480	67	0.6
**Deaths**	0	0	0
**SMR**			
**DALY**	232	32	0.23
**YLD**	104	15	0.13
**YLL**	129	18	0.16
**Cases**	14	2	0.02
**Deaths**	7	1	0.01

DALY = Disability-Adjusted Life Years (mortality and morbidity); YLL = Years of Life Lost (mortality); YLD = Years Lived with Disability (morbidity); MMR = Mild Mental Retardation; SMR = Severe Mental Retardation

### Sensitivity analyses

**Fig 3:**
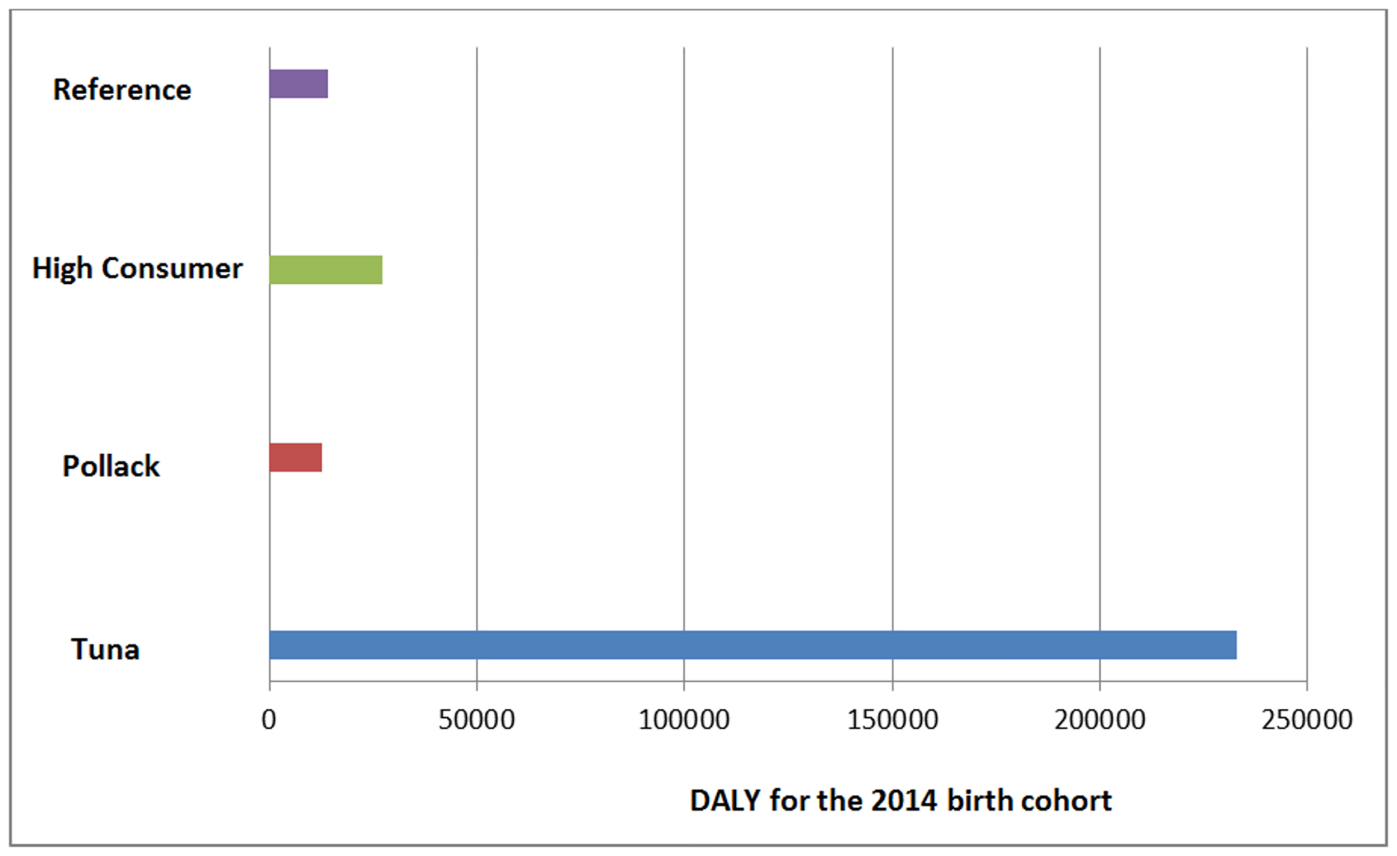
Results of the scenario analysis. Disease burden of the 2014 birth cohort in Germany for high fish, only pollack and only tuna consumers.

Fig 3 shows the overall results for MMR and SMR for the three scenarios. The highest burden of 233,118 DALY (2.5th percentile 227,374 DALY, 97.5th percentile 238,918 DALY) was computed in the tuna scenario. Instead of the 494 cases of mental retardation computed in the reference scenario (Table 2), here over 8000 cases were calculated. The lowest burden of 12,468 DALY were computed in the pollack scenario (2.5th percentile 11,250 DALY, 97.5th percentile 13,697 DALY). This burden is on average 12% lower than in the reference scenario. However, the high consumer scenario produced more than twice as much burden as the reference scenario (27,319 DALY, 2.5th percentile 25,574 DALY, 97.5th percentile 29,067 DALY).

**Fig 4:**
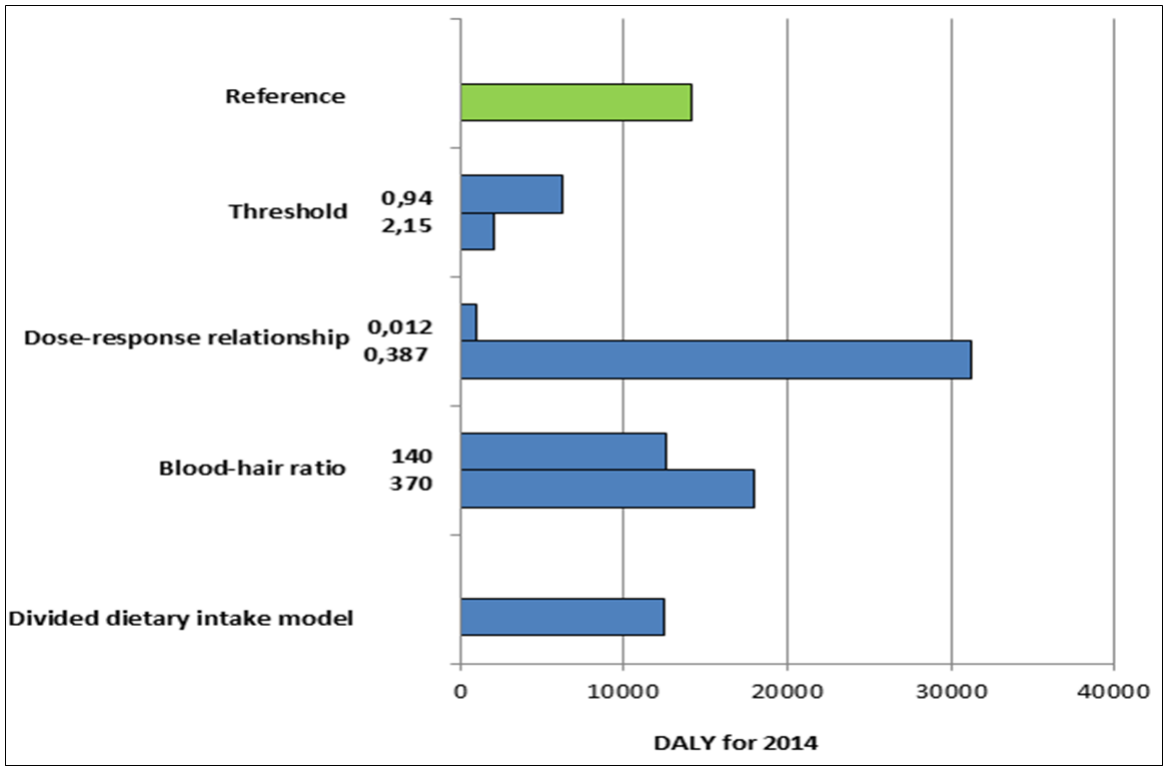
Results of the sensitivity analysis. Disease burden of the 2014 birth cohort in Germany for different uncertainty parameters.

In our additional sensitivity analysis, we addressed the uncertainty in: 1) the threshold values of critical maternal hair methylmercury concentrations, 2) dose-response relationships and 3) blood-hair conversion factors. The greatest deviation to the reference scenario occurred by changing the dose-relationship between maternal hair concentration and IQ point decrease. Fig 4 shows the results for the decrease of 0.012 and 0.387 IQ points per μg/g mercury in hair. Furthermore, the results show the strong DALY reduction due to a threshold of 0.94 and 2.15μg/g mercury in hair. Similar results, which are approximately 12% lower than in the reference scenario, demonstrate a change of the blood-hair conversion factor from 250 to 140 and the divided dietary intake model output by 14.

## Discussion

In this study, we calculated for the first time, the disease burden caused by methylmercury with the DALY method for Germany. For this purpose, we used empirical data for 2014 (birthrate, life expectancy and sale volume for fish species) and the measured weight of pregnant women, the amount of fish consumption and the methylmercury concentration of the consumed fish species. Our most important result is that the disease burden was predominantly caused by the consumption of fish products with high methylmercury contamination (e.g. tuna) by pregnant women. A huge majority of the DALYs was attributed to morbidity (99%) compared to mortality. Of the total disease burden (14,186 DALY), 13,954 DALY were due to MMR. The remaining disease burden was a result of SMR with equal proportions of premature death and morbidity.

We know only of one further study from the Netherlands, in which the disease burden due to methylmercury exposure by fish consumption was calculated [10]. This risk-benefit study focused on two scenarios with a weekly fish intake of either 200g or 500g and the health outcome IQ change. For the IQ change, 8 and 25 DALY per 100,000 inhabitants were computed respectively. Our calculations resulted in an average of 18 DALY per 100,000 inhabitants, although the pregnant women in Germany eat on average less fish than those considered in the study from the Netherlands. However, we calculated a higher exposure with an average exposure of 0.13μg/g and a standard deviation of 0.05 μg/g, while Hoekstra et al. (2013) used a lower and fixed value of 0.05 μg/g for the exposure [10]. It may be possible, that we overestimate the exposure of methylmercury, because there are no data on the fish species preferred by pregnant women, only a few data on the methylmercury concentration in fish and no information on the methylmercury concentration of some species (e.g. zander, haddock) [3, 11]. Furthermore, it is possible that pregnant women followed current dietary recommendations, which advise not to consume fish with high methylmercury concentrations like tuna or shark frequently [37].

The WHO recommendations for calculating the disease burden of methylmercury contains estimations for different subgroups and the health outcome MMR. Our exposure results are slightly higher than the lowest WHO example of female Canadian sport fishers [7]. The sport fishers had a mean hair mercury level of 0.68μg/g. Despite similar exposure, the calculated burden in our study is three times higher (7.2 vs. 19.8 DALY per 1,000 newborns). The main reason for this difference, is the use of age weighting and discounting in the WHO example [7]. The age-weighting gave more importance to the middle age group. For example, a DALY in this age group leads to more burden than a DALY in the infancy. The discounting is adopted from the economy and based on the concept that persons prefer benefits immediately rather than in the future. Under this assumption the WHO example used a discount rate of 3% [27]. Discounting leads to a strong reduction of DALY in lifelong diseases. After further development of the DALY concept, age-weighting and discounting are only rarely used, so we did not apply them here [38].

The exposure from the WHO examples is measured in hair of female Canadian sport fishers, who fished in the St. Lawrence River. The river is known for fishes with high mercury concentrations [39]. It is likely, that the exposure of the sport fishers is higher than the exposure of the pregnant fish consumer in Germany. Because we had no data for the hair concentration of pregnant women in Germany, we used the dietary intake model with consumption data to convert the fish consumption into hair concentration levels. There are indications, that the model overestimates the mercury concentration in hair. Canuel et al. (2006) compared mercury hair concentrations predicted by a model similar to the dietary intake model used here, with measured mercury hair levels in three regions. They found in two regions a slight overestimation and in one region an actual hair concentration 14 times less than predicted. Based on these results, they conclude that the dietary intake model may not be an accurate indicator of the mercury exposure in the population, because it cannot comprehensively represent the individual variability in absorption and elimination rates [35]. This finding led us to divide the dietary intake model output by 14 in our uncertainty analysis. This resulted in a reduced disease burden of 12,441 DALY, but did not affect our results of the proportion of morbidity and mortality and the critical role of fish species with high methylmercury contamination.

In the European human biomonitoring pilot study DEMOCOPHES hair samples of 120 mother and child pairs from Germany were taken. The samples of the mothers were tested for mercury and other contaminants. So far, only the average mercury concentration of 0.113 μg/g was published [40]. Our computed hair concentration is almost eight times higher than these measured values. A lower measured value in the pilot study was to be expected, because this study included female fish- and non-fish consumers, whereas our study only included women, who consume fish. After including the non-fish consumers in our model, the hair concentration decreased by 10%. Furthermore, the NVS II data showed that pregnant women consume more often and higher amounts of fish than non-pregnant women [19]. However, the pilot study is not representative. The sample size was very small and all tested mothers come from one region. Thus, mothers from regions close to the sea, where a higher level of fish consumption is expected, are not included [41]. Unfortunately, there are no more recent human biomonitoring data. The last available data for adults are published in the German Environmental Survey 1998. For men and women (18–69 years) an average blood concentration of 0.58μg/l were measured [42]. Our results are higher, what is not remarkable because the non-fish consumers are included. Furthermore, the occurrence of mercury in the environment and the fish eating habits in the population could have changed in the last years.

Since the available fish consumption data for pregnant women are based only on 82 pregnant women, we performed a scenario analysis for the high consumers (85.4g fish and seafood per day) to assess how much the disease burden would increase with higher fish consumption. Our result showed for the high consumer scenario 27,319 DALY. A value, which is more than twice as much as in the reference scenario. For more accurate disease burden estimations, more data relating to the consumed fish species and the amount of fish consumption from pregnant women are needed.

In our uncertainty analysis, we found that the greatest deviation occurred by changing the dose-response relationship between the measured maternal hair value and the IQ level decrease of their infants. Because Axelrad et al. (2007) included only three epidemiological studies to determine the dose-response relationship, the WHO recommended uncertainty analysis with the values of the 95% confidence interval [7, 8]. Using these values, we received a minimal disease burden of 930 DALY and a maximum of 31,218 DALY (Fig 4). Axelrad et al. (2007) performed sensitivity analysis for the dose-response relationship and calculated an IQ point decrease between 0.13 and 0.25 IQ points for each μg/g of maternal hair mercury [8]. Based on this additional analysis, we assume that the decrease of 0.18 IQ points for each μg/g of maternal hair mercury is a valid value.

Until now, only a few studies investigate the disease burden of chemical contaminants and food of animal origin. This makes a ranking of the health impact difficult. For Germany, a disease burden study estimated 46.6 DALY per 100,000 inhabitants caused by dioxin in food [43]. This result is significantly higher than our computed disease burden of methylmercury (18 DALY per 100,000 inhabitants). We suppose that the chosen health outcome is an important factor for the difference, because Hänninen et al. (2011) dealt with the health outcome of all types of cancer and assumed that all cases are fatal [43]. Furthermore, the previously cited Dutch risk-benefit analysis estimated the disease burden of dioxin via fish intake. Also this study estimated a higher dioxin-caused burden with the health outcomes decrease of sperm count and thyroid hormone production (200g scenario: 22 DALY, 500g scenario: 60 DALY per 100,000 inhabitants) than by methylmercury [10]. These results suggest that dioxin in food of animal origin leads to a higher disease burden than methylmercury.

Our predicted hair and blood values are higher than measured reference values. Because of a lack of data for preferred fish species of pregnant women, the fish exposure and recent biomonitoring data, it is possible, that we overestimate the disease burden of methylmercury.

With our study, we show a possibility to calculate the disease burden of contaminants with high natural variability illustrated by the example methylmercury. Better estimates of positive and negative effects can also be achieved by the use of biomonitoring data and data on the fish consumption habits of pregnant women. With these, a risk-benefit-analysis for fish would be feasible. Our current estimation considers the adverse effects of fish consumption exclusively. Besides methylmercury, fish also contains many healthy contaminants like omega-3 fatty acids, iodine or selenium. These ingredients are associated with positive effects like allergy prevention, IQ gain or prevention of cardiovascular diseases [10, 44]. Fish consumption in pregnancy appears to be useful under these conditions. To quantify these positive aspects, further research on the benefits of fish consumption is required.

## Supporting information

S1 Table Relative consumption of fish species and MeHg-content data used by the beta-PERT distribution. (DOCX)

S2 Table Distribution functions of parameters used to estimate the health burden of methylmercury. (DOCX)

S1 Fig DALY distribution. Distribution of overall methylmercury-associated disease burden measured in terms of disability-adjusted life years (DALYs) of the 2014 birth cohort in Germany.
(TIF)

S2 Fig Distribution of the methylmercury-induced mortality and morbidity. Percentage of mortality (Years of Life Lost YLL) and morbidity (Years Lived with Disability YLD) on the total disease burden, the burden of severe mental retardation (SMR) and mild mental retardation (MMR).
(TIF)
