# Supplementary material for: Disease burden of methylmercury in the German birth cohort 2014
Source: PLoS One. 2018 Jan 11;13(1):e0190409. doi: 10.1371/journal.pone.0190409 (PMC5764270; doi:10.1371/journal.pone.0190409)
Supplement: S1 Table — (DOCX) [file pone.0190409.s001.docx]

| **Fish species** | **Relative consumption of fish species in Germany (%) [1]** | **Total number of tested fish**  **[2, 3]** | **P for the beta-PERT distribution** | | | | **Comment** |
| --- | --- | --- | --- | --- | --- | --- | --- |
|  |  |  | **Minimum** | **Median** | **Maximum** | **Reference** |  |
|  |  |  | **MeHg in μg/g** | | |  |  |
| Salmon | 22 | 1 | 0.0279 | 0.028 | 0.0281 | [2] |  |
| Pollack | 21.4 | 122 | 0.001 | 0.007 | 0.048 | [2] |  |
| Herring | 14.7 | 205 | 0.007 | 0.022 | 0.289 | [2, 3] |  |
| Tuna | 12.4 | 125 | 0.031 | 0.218 | 2.265 | [3] |  |
| Trout | 5.9 | 15 | 0.029 | 0.048 | 0.072 | [3] |  |
| Catfish | 2.9 | 1 | 0.0059 | 0.006 | 0.0061 | [4] | Hg-value matched with MeHg |
| Cod | 2.7 | 58 | 0.01 | 0.151 | 0.412 | [2] |  |
| Mackerel | 2 | 206 | 0.006 | 0.057 | 0.238 | [2, 3] |  |
| Coalfish | 1.5 | 23 | 0.01 | 0.07 | 0.488 | [3] |  |
| Redfish | 1.4 | 152 | 0.019 | 0.103 | 0.698 | [2, 3] |  |
| Plaice | 1.2 | 159 | 0.009 | 0.03 | 0.228 | [2, 3] |  |
| Zander | 1 | - | 0.019 | 0.103 | 0.698 | [2, 3] | MeHg-valuesfromredfish |
| Carp | 0.8 | 16 | 0.008 | 0.030 | 0.106 | [3] |  |
| Haddock | 0.7 | - | 0.006 | 0.034 | 0.182 | [2, 3] | MeHg-valuesfromcod |
| Sardine | 0.7 | - | 0.007 | 0.027 | 0.289 | [2, 3] | MeHg-valuesfromherring |
| Tilapia | 0.5 | - | 0.019 | 0.103 | 0.698 | [2, 3] | MeHg-valuesfromredfish |
| Devilfish | 0.5 | - | 0.019 | 0.103 | 0.698 | [2, 3] | MeHg-valuesfromredfish |
| Hake | 0.3 | - | 0.006 | 0.03 | 0.182 | [2, 3] | MeHg-valuesfromcod |
| Other | 7.4 | - | 0.001 | 0.045 | 2.265 |  | Minimum, median and maximum from all fish species |

1. Fischinformationszentrum e.V., *Fischwirtschaft: Daten und Fakten*. 2016: Hamburg.

2. Kruse, R., Behrens, S., Sommerfeld, C., Bartelt, E., *Klimawandel bedingte Aufnahme von toxischem Methylquecksilber über den Fischkonsum*, in *Umweltforschungsplan des Bundesministeriums für Umwelt, Naturschutz und Reaktorsicherheit*, I.f.F.u.F. Niedersächsisches Landesamt für Verbraucherschutz und Lebensmittelsicherheit, Cuxhaven, Bundesinstitut für Risikobewertung, Berlin, Editor. 2008: Berlin. p. 76.

3. Kruse, R., Bartelt, S., *Exposition mit Methylquecksilber durch Fischverzehr und Etablierung analytischer Methoden zur Bestimmung von Methylquecksilber in Fischereierzeugnissen*, in *Umweltforschungsplan des Bundesministeriums für Umwelt, Naturschutz und Reaktorsicherheit*, I.f.F.u.F. Niedersächsisches Landesamt für Verbraucherschutz und Lebensmittelsicherheit, Editor. 2008: Cuxhaven. p. 30.

4. Bundesamt für Verbraucherschutz und Lebensmittel, *Berichte zur Lebensmittelsicherheit 2011: Monitoring*. 2011: Berlin.
