# Supplementary material for: Disease burden of methylmercury in the German birth cohort 2014
Source: PLoS One. 2018 Jan 11;13(1):e0190409. doi: 10.1371/journal.pone.0190409 (PMC5764270; doi:10.1371/journal.pone.0190409)
Supplement: S2 Table — (DOCX) [file pone.0190409.s002.docx]

| **Health outcome** | **Model parameters** | **Input distributions** |
| --- | --- | --- |
| MMR | Incidence per 1,000 newborns, 2014 | Normal (0.7529; 0.035) |
|  | Duration (years)  male  female | Fixed (78.13)  Fixed (83.05) |
|  | Disability Weight | Fixed (0.361) |
| SMR | Incidence per 1,000 newborns, 2014 | Normal (0.0218; 0.0058) |
|  | Duration (years)  male  female | Fixed (78.13)  Fixed (83.05) |
|  | Disability Weight | Beta-PERT (0.041; 0.098; 0.123) |
|  | Premature mortality | Uniform (0; 0.0218) |
|  | Average age at death  male  female | Beta-PERT (58.6; 62.5; 78.13)  Beta-PERT (58.6; 66.44; 83.05) |
