# Supplementary figures and images for: Disease burden of methylmercury in the German birth cohort 2014
Source: PLoS One. 2018 Jan 11;13(1):e0190409. doi: 10.1371/journal.pone.0190409 (PMC5764270; doi:10.1371/journal.pone.0190409)

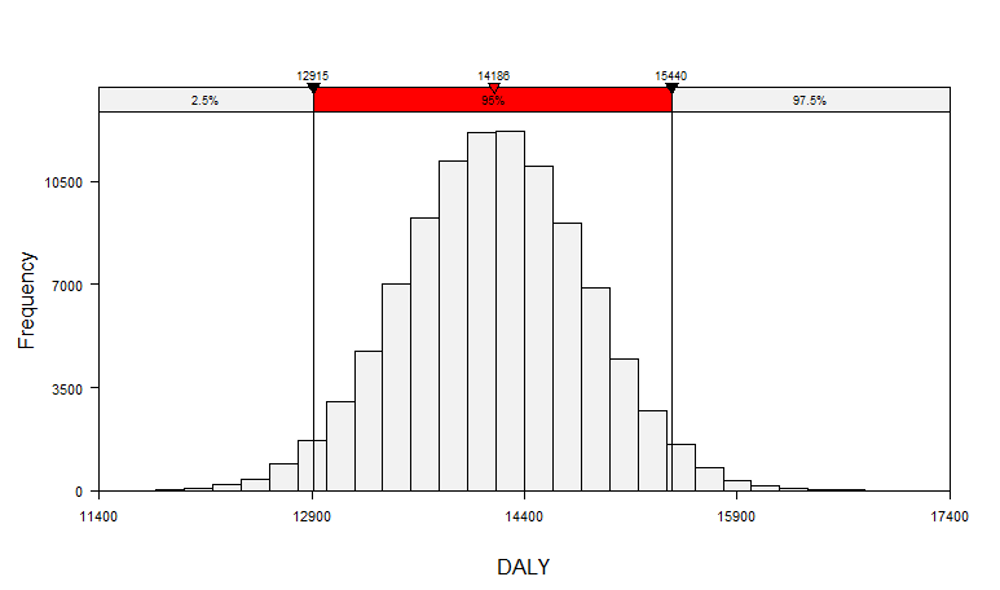

Supplement: S1 Fig — Distribution of overall methylmercury-associated disease burden measured in terms of disability-adjusted life years (DALYs) of the 2014 birth cohort in Germany. (TIF) [file pone.0190409.s003.tif]

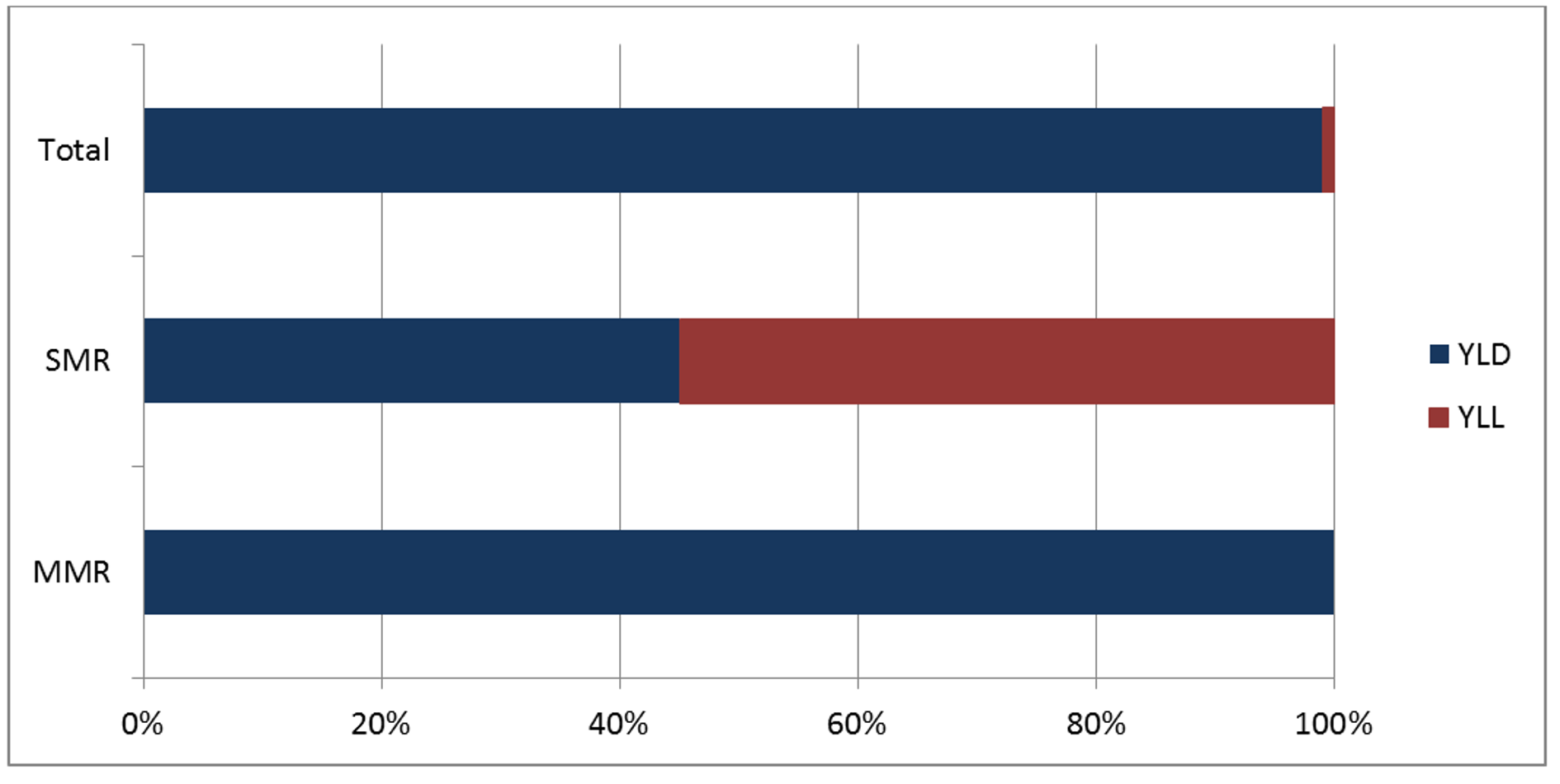

Supplement: S2 Fig — Percentage of mortality (Years of Life Lost YLL) and morbidity (Years Lived with Disability YLD) on the total disease burden, the burden of severe mental retardation (SMR) and mild mental retardation (MMR). (TIF) [file pone.0190409.s004.tif]
